# How to study the role of volunteer responders in the chain of survival

**DOI:** 10.1007/s12471-022-01749-w

**Published:** 2022-12-20

**Authors:** Remy Stieglis, Rudolph W. Koster, Arthur A. M. Wilde

**Affiliations:** 1 Department of Cardiology, Amsterdam UMC, location AMC, University of Amsterdam, Amsterdam, The Netherlands; 2 Amsterdam Cardiovascular Sciences, Heart Failure and Arrhythmias, Amsterdam UMC, location AMC, University of Amsterdam, Amsterdam, The Netherlands

Dear Editor,

Recently, Oosterveer et al. [1] reported in the* Netherlands Heart Journal* on the association between a volunteer responder system and survival outcomes. We welcome any report on resuscitation outcome that confirms or helps to improve outcomes in out-of-hospital cardiac arrest. Unfortunately, this study has several significant methodological issues that in our opinion do not allow any conclusion to be made. We focus on the main issues.

First, in the present study controls are derived from a previously published study performed between 2011 and 2013 [2] and the cases (with text message (TM) responders added) were resuscitated in 2018. But, in contrast to the historic controls, not all cases are included. Those in which an automated external defibrillator (AED) delivered a defibrillation shock followed by return of spontaneous circulation (ROSC) before the emergency medical service (EMS) arrived were excluded because the EMS did not need to continue cardiopulmonary resuscitation (CPR). This may occur in 11–13% of cases with the best possible prognosis but appears to have been excluded from this analysis! [3] Also, EMS-witnessed cases—about 10% in each group in this study—should not be included, as in these cases first responders and TM responders would never be alerted. Finally, unlike the control group, in the TM group only patients taken to Leiden University Medical Centre were included in the survival analysis. About 25% of the patients were transferred to another hospital without intervention capabilities, and survival data were missing for most of those cases. If patients are first taken to a hospital without intervention capacity, this might delay intervention, possibly with a negative impact on survival.

Second, the statistical analysis. The variables ‘CPR before ambulance arrival’ and ‘AED before ambulance arrival’ were entered into the model as covariates. These variables are the most important process outcomes of a TM responder system. If these variables are used as a covariate in the model, its effect is ‘controlled away’.

In the multivariate regression analysis ‘First monitored rhythm shockable’ was the reference group [odds ratio (OR) = 1], but the presentation is confusing: Table 2 mentions an OR for shockable rhythm of 0.22–0.21, but that is the OR for *non*-shockable rhythms. Similarly, this is the case for witnessed arrest and use of the Lund University Cardiopulmonary Assist System (LUCAS). LUCAS use was also defined as a reference group and had an OR of 10–3.3, while use of mechanical chest compression devices is often associated with an OR < 1, an example of ‘resuscitation time bias’ [4]. In the Discussion section, the authors explain why a negative association of witnessed arrest and shockable initial rhythm on survival was found. The authors are mistaken here: Table 2 incorrectly shows the opposite ORs for these variables, because the chosen reference group is incorrectly presented, as explained above. This ‘solves’ the discussed issue.

The main conclusion of this study is that a volunteer responder system is associated with a higher percentage of patients achieving ROSC, but not with survival. The study, however, suffers from significant methodological flaws in design and analysis, which preclude acceptance of this conclusion.
